# Lessons learned from implementation of four HIV self-testing (HIVST) distribution models in Zambia: applying the Consolidated Framework for Implementation Research to understand impact of contextual factors on implementation

**DOI:** 10.1186/s12879-024-09168-5

**Published:** 2024-03-06

**Authors:** Musonda Simwinga, Lwiindi Gwanu, Bernadette Hensen, Lucheka Sigande, Mwami Mainga, Thokozile Phiri, Eliphas Mwanza, Mutale Kabumbu, Chama Mulubwa, Lawrence Mwenge, Chiti Bwalya, Moses Kumwenda, Ellen Mubanga, Paul Mee, Cheryl C. Johnson, Elizabeth L. Corbett, Karin Hatzold, Melissa Neuman, Helen Ayles, Miriam Taegtmeyer

**Affiliations:** 1 Zambart, Lusaka, Zambia; 2 Clinical Research Department, Faculty of Infectious and Tropical Diseases, London School of Hygiene and Tropical Medicine, London, UK; 3 Society for Family Health (SFH), Lusaka, Zambia; 4 Malawi Liverpool Wellcome Trust Clinical Research Programme, Blantyre, Malawi; 5 National HIV/AID/STI/TB Council (NAC), Lusaka, Zambia; 6 Department of Infectious Disease Epidemiology, Medical Research Council International Statistics and Epidemiology Group, London School of Hygiene and Tropical Medicine, London, UK; 7 Global HIV, Hepatitis and STI Programmes, World Health Organisation, Geneva, Switzerland; 8 Population Services International, Johannesburg, South Africa; 9 Department of Clinical Sciences, Liverpool School of Tropical Medicine, Liverpool, UK; 10 Tropical Infectious Diseases Unit, Liverpool University Hospitals Foundation Trust, Liverpool, UK; 11 Department of Public Health, Sexual and Reproductive Health Group, Institute of Tropical Medicine, Antwerp, Belgium

**Keywords:** HIV self-testing (HIVST), Community-led, Workplace, Secondary distribution, Contextual factors, Zambia

## Abstract

**Background:**

Although Zambia has integrated HIV-self-testing (HIVST) into its Human Immunodeficiency Virus (HIV) regulatory frameworks, few best practices to optimize the use of HIV self-testing to increase testing coverage have been documented. We conducted a prospective case study to understand contextual factors guiding implementation of four HIVST distribution models to inform scale-up in Zambia.

**Methods:**

We used the qualitative case study method to explore user and provider experiences with four HIVST distribution models (two secondary distribution models in Antenatal Care (ANC) and Antiretroviral Therapy (ART) clinics, community-led, and workplace) to understand factors influencing HIVST distribution. Participants were purposefully selected based on their participation in HIVST and on their ability to provide rich contextual experience of the distribution models. Data were collected using observations (*n* = 31), group discussions (*n* = 10), and in-depth interviews (*n* = 77). Data were analyzed using the thematic approach and aligned to the four Consolidated Framework for Implementation Research (CFIR) domains.

**Results:**

Implementation of the four distribution models was influenced by an interplay of outer and inner setting factors. Inadequate compensation and incentives for distributors may have contributed to distributor attrition in the community-led and workplace HIVST models. Stockouts, experienced at the start of implementation in the secondary-distribution and community-led distribution models often disrupted distribution. The existence of policy and practices aided integration of HIVST in the workplace. External factors complimented internal factors for successful implementation. For instance, despite distributor attrition leading to excessive workload, distributors often multi-tasked to keep up with demand for kits, even though distribution points were geographically widespread in the workplace, and to a less extent in the community-led models. Use of existing communication platforms such as lunchtime and safety meetings to promote and distribute kits, peers to support distributors, reduction in trips by distributors to replenish stocks, increase in monetary incentives and reorganisation of stakeholder roles proved to be good adaptations.

**Conclusion:**

HIVST distribution was influenced by a combination of contextual factors in variable ways. Understanding how the factors interacted in real world settings informed adaptations to implementation devised to minimize disruptions to distribution.

## Introduction

In 2018, approximately 25% of people living with HIV in Zambia were unaware of their status and only 64% of women and 52% of men aged 15–59 had tested for HIV in the last 12 months [1]. HIV self-testing (HIVST), a process where an individual collects their own specimen, conducts an HIV test and interprets the result [2], is recommended by the World Health Organization (WHO) [3]. Zambia has since embraced the potential of HIVST to increase HIV testing coverage in populations underserved by existing testing services, including adolescents, men and key populations [4–8]. HIVST has been shown to be acceptable, empowering and easy-to-use, offering users control over the testing process [2, 9–13]. As part of the STAR (Self-testing Africa) Initiative [14–16], studies in Zambia have shown that HIVST is acceptable but overall awareness and use remains low at population-level [17] and the uptake of HIVST and impact on knowledge of HIV status varies across populations and distribution models [18–21]. Despite national-level integration of HIVST, including within Zambian regulatory frameworks and HIV testing strategies, [19, 20, 22], evidence demonstrating how to optimally distribute HIVST to achieve high testing coverage in Zambia remains limited.

Multiple factors (context) usually surround implementation of an intervention [23]. Contextual factors, including strong leadership [24, 25], buy-in from target community and stakeholders, and simplicity of the intervention [24], are important when implementing and scaling-up interventions, including HIVST interventions. Other factors include organisational support, financial resources, and social relations and support [25]. However, interventions are often complex, with core intervention elements and contextual factors interacting at different levels. Variability in implementation arising from contextual differences makes it hard for interventions to be brought to scale [23]. However, understanding the interplay between the factors in one context is a necessary step to determining what adaptations might be required for an intervention to replicate results in another context. For example, significant modifications to evaluated HIVST distribution models may have to be made for HIVST to produce similar results in different contexts. However, these contextual factors need to be identified and understood; first to be able to determine the appropriate combination of fidelity (whether the models are being delivered as intended), dose (the quantity of aspects of the models being implemented) and adaption for the HIVST distribution models to optimise uptake [26].

We conducted a prospective evaluation of four HIVST distribution models to explore contextual factors affecting implementation of HIVST distribution models in Zambia, using a qualitative case study approach. These models included secondary distribution to male partners of pregnant women through antenatal care (ANC) and secondary distribution to partners of people with HIV at Antiretroviral Therapy (ART) clinics, as well as community-led, and workplace distribution models. In this paper, we identify and describe aspects of context that facilitate or impede implementation of these distribution models, and we use findings to inform contextual factors that need to be considered in other places in Zambia and similar countries that plan to implement these models.

## Methods

Evaluation of the distribution models was done between November 2018 and July 2019, and between September 2019 and March 2020 for the second phase of the workplace model (Table 1).

**Table 1:** Timing and location of HIVST distribution models

Distribution model	Distribution method	Location	Distribution period	Evaluation period
Model 1: Secondary distribution (one ANC clinic)	Pregnant woman given 1 HIVST kit to deliver to primary male partner	Lusaka	June 2018-Dec 2019	November 2018—July 2019
Model 2: Secondary distribution (one ART clinic)	Individual with HIV given 1 HIVST kit to deliver to a partner	Lusaka	June 2018-Dec 2019	November 2018—July 2019
Model 3: Community-led	Individual given 1 HIVST kit by shop owner	Lusaka	June 2018-Dec 2019	November 2018—July 2019
Model 4: Workplace distribution- phase 1 +	Worker given 1 HIVST kit by a workplace champion (optional: 2 kits for self and partner)	Lusaka, government, institutions	June 2018-Dec 2019	November 2018 – July 2019
		Lusaka, private institutions	June 2018-Dec 2019	November 2018 – July 2019
Model 4: Workplace distribution- phase 2 +	Worker given 1 HIVST kit by a workplace champion (optional: 2 kits for self and partner)	Copperbelt, Mine (1)	Sept – Dec 2019^a^	September–October, 2019
		Lusaka, manufacturing company (1)	July – Dec 2019^a^	February–March, 2020

^a^Distribution continued beyond this date. + Phase 1 distribution was done in Lusaka only in both government and private institutions, while phase 2 was done in both Lusaka and Copperbelt in a manufacturing company and mine respectively

### Study location

The four models were implemented in urban settings. The two secondary distribution models and the community-led model were implemented in Lusaka, in one community and a linked health facility. The workplace model was implemented in Lusaka and Copperbelt provinces (Table 1).

### The HIVST distribution models

The Ministry of Health (MOH), with support from Society for Family Health (SFH), distributed HIVST between June 2018 and December 2019. The four models were selected for evaluation because they were identified and recommended by MOH as the models with the most potential to reach populations underserved by existing HIV testing services.

All four models had common, core elements supported by SFH and MOH, namely, recruitment and training of distributors, quality assurance of test kit storage, stock control, and data collection. The identification and training of distributors for the community-led model was led by community representatives while that for secondary distribution was done by SFH, health facility staff and community representatives. Management of institutions identified individuals to train as workplace champions. Implementing partners included MOH through head office and the provincial and district health offices, SFH, National AIDS Council (NAC) and International Labour Organisation (ILO). The involvement of multiple stakeholders provided the necessary interaction with the distributors early on and was part of the buy-in process.

All distributors were trained on how to demonstrate use of an HIVST kit; use of referral slips and recording client details (age, sex, testing history). Distributors were required to participate, to some extent, in supply chain management and write progress reports.

#### Model 1: secondary distribution through ANC clinic

This model was designed to reach male partners of pregnant women attending ANC. At the start of the day, volunteer distributors would give a health talk as women waited to be attended to followed by a demonstration of HIVST. The distribution of HIVST kits occurred in a designated private room, conveniently located near the entrance of the facility. On average, two female distributors (out of the four assigned), aged 20–40 were present at the ANC daily. They distributed one HIVST kit to women who accepted an offer of HIVST, or two to women who wanted to test with their partners.

#### Model 2: secondary distribution through ART clinic

This model was designed to target untested (or previously negative) partners of HIV positive patients on treatment. The ART clinic was conveniently located near the entrance to the health facility. The health talks and HIVST demonstrations happened in a shelter outside the ART clinic. Some clients listened to the health talks from inside the clinic corridors when the shelter was full. Unlike the ANC clinic, ART clients came at different times. Those who came after the health talk were not offered the HIVST. Two distributors (a man and woman) were expected to be always available; one to attend to clients in the corridors and the other to assist the pharmacist in distributing HIVST kits, and to provide health talks. Distribution of HIVST kits, was done at the pharmacy as the clients collected drugs as well as in the counselling room or corridors when too many clients turned up.

Distributors in both secondary distribution models received non-financial incentives such as t-shirts and caps, albeit intermittently, from the health facility management for volunteering at the facility, and not for distributing HIVST kits.

#### Model 3: community-led distribution

This model targeted individuals, mainly men, who do not commonly access HIV testing at the health facility. The criteria for identifying distributors and distribution points, and their selection, was done by the Health Centre Committee (HCC), a community-based organisation, created by an Act of Parliament. Some HCC members were selected as distributors. Distribution was primarily through small makeshift shops known as ‘*kantemba*’—(plural *tuntemba). Tuntemba* were selected on the basis that the shop was easily accessible, located in high density areas and traded in non-alcoholic substances. Owners were expected to be ‘mature’, trusted in the community, with basic reading and writing skills, and willing to work without payment. Trained *tuntemba* owners introduced HIVST to clients, demonstrated how to use HIVST kits, distributed one kit per client, and responded to questions. Kits were replenished from the linked health facility implementing secondary distribution in a draw down model. *Tuntemba* owners were compensated with K50 ($3) per month for airtime and a K200 ($11) monthly transport refund for movements to and from the health facility. They received additional material incentives in the form of promotional material like branded T-shirts, caps, and bags. Promotion of HIVST was done using brochures, danglers, and posters in the community. A community-based model using Community Based Distribution Agents (CBDAs) was implemented alongside the community-led model.

#### Model 4: workplace distribution

This model was designed to target men, thus workplaces with a large male workforce were prioritised. In the first phase, HIVST was distributed in private companies and government institutions in Lusaka by SFH-trained peer educators with help from trained workplace champions, peer educators or wellness programme staff. Evaluation of the distribution in the second phase was done in one mine on the Copperbelt, and one manufacturing company in Lusaka, as requested by the MOH, NAC, WHO and ILO. Trained workplace champions conducted sensitization meetings with employees incorporated into routine activities and communication channels such as safety meetings, lunch time meetings, scheduled campaigns, and on events such as the World AIDS Day. In the Lusaka-based company, distribution was done from the company clinic by two clinical officers, trained as workplace champions, and supported by five peer educators. In the mine, distribution was done by one workplace champion from the mine’s training school, some distance from the mine. The champions distributed one HIVST kit per individual worker, but workers who consented to get for their partners received two kits. Workplace champions did not receive monetary or material incentives.

### Data collection

We used the qualitative case study method to gain in-depth understanding of the four HIVST distribution models. Case studies are suitable for exploring complex situations by collecting multiple perspectives with the use of multiple data collection methods, employing the ‘how’ and ‘why’ questions [27]. This approach enabled us to understand contextual factors affecting implementation of the distribution models [28]. The multiple methods used included: community mapping, to document historical and structural features that might influence distribution [29], observations of distribution, to obtain insights into the process of HIVST distribution [30], in-depth Interviews (IDIs), to explore perspectives and experiences of HIVST [31], and focus group discussions (FGDs) to explore collective insights [32] about what worked and what did not work well with the community-led and secondary distribution models (see Table 2).

**Table 2:** Qualitative research evaluation activities

	**Data collection activity**	**HIVST distribution models**				
		**Secondary distribution**		**Community-led**	**Workplace**	**Total**
		**ANC**	**ART**			
1	Community Mapping and spiral walk	0	0	1	0	1
2	Observation of training	0	0	0	2	2
3	Observation of HIVST distribution	9	5	5	10	29
4	IDIs with distributors and health care workers (HCWs)	1	2	7	2	12
5	IDIs with users—(index, workers)	21	11	0	18	50
6	IDIs with community users	0	0	9	0	9
7	IDIs with SFH and workplace staff	0	0	3	1	4
8	IDI with HCC member	0	0	1	0	1
9	IDI with district AIDS coordinating advisor	0	0	0	1	1
10	FGD with community members	0	0	2	0	2
11	FGD with community-based distributors	0	0	1	0	1
12	FGD with ANC participants	3	0	0	0	3
13	FGD with ART participants	3	0	0	0	3
	**Totals**	**37**	**18**	**29**	**34**	**118**

Process evaluation of the distribution models was conducted in part by reviewing monitoring and evaluation data. In this paper we will present descriptive results of the number of kits distributed, % distributed by model and age of those accessing the kits for the secondary distribution models.

Ninety-four (94) participants took part in the FGDs. Of these, 57 (61%) were women and 37 (39%) were men (see Table 3). The average age of the participants was 38 years. The youngest was 18 years and the oldest was 74 years. FGD participants were not considered for IDIs.

**Table 3:** FGD participants

	**Men (%)**	**Women (%)**	**Total**
**FGDs (totals)**	**37 (39)**	**57 (61)**	**94**
Mapping	3 (30)	7 (70)	10
ART participants	16 (50)	16 (50)	32
ANC participants	0 (0)	23 (100)	23
Community based distributors	5 (45)	6 (55)	11
Community members	13 (72)	5 (28)	18
**Demographic characteristics**			
18–24 years	6 (25)	18 (75)	24
25–35 years	7 (44)	9 (56)	16
36–50 years	12 (34)	23 (66)	35
Over 50	12 (63)	7 (37)	19

We used the maximum variation principle to purposively select participants based on the HIVST distribution model implemented; their role; participants’ ability to provide rich context specific information on the model of interest; and the need to attain a mix of age, gender, and social status.

Data were collected by four trained social science research assistants using English and two local languages, Bemba, and Nyanja. The community mapping FGDs with HCC members explored the history of the community, and community features likely to influence HIVST uptake for the community-led and secondary distribution models. This was followed by a spiral walk during which the location of the health facility and distribution points were visualized in relation to different features including shops, houses, and road networks on the map. Observations of active distribution points were conducted using a checklist that covered different aspects of the process of HIVST distribution in each setting. IDI topic guides covered issues around benefits, challenges, and feasibility of implementing the models. IDIs with staff focused on the process of planning, establishing, and monitoring HIVST distribution. Mixed FGDs with community members, community-based distributors and with ANC and ART participants complemented findings from IDIs and explored enablers and barriers to distribution for the community-led and secondary distribution models. All IDIs and FGDs were audio recorded. The IDIs and FGDs audio recordings were transcribed verbatim, and those conducted in local language were translated during transcription. Social science research assistants produced written summaries for each interview.

### Data analysis

Data analysis was informed by thematic analysis approach drawing on emerging issues from the data (inductive) and from existing literature including the Consolidated Framework for Implementation Research (CFIR) domains (deductive), with particular focus on factors affecting intervention implementation. The CFIR is an implementation science framework developed to guide systematic assessment of potential barriers and facilitators (contextual factors) of evidence-based interventions. It is made up of 39 constructs divided into five domains [25, 33]. A small sample of transcripts and summaries were purposefully selected and used to develop themes (inductively and using the CFIR domains and constructs) and a coding framework, which was applied across all data sets. Data were inputted into and managed by ATLAS.ti. Themes were then organised in matrixes and the latter populated as the research assistants read and re-read the transcripts. This process benefited from prior data analysis frameworks developed by the STAR Qualitative Research Network^1^ [34]. To improve the trustworthiness of the data, two senior social scientists held regular debriefing meetings with the research assistants, and checked summaries for detail, errors, and completeness.

We used four of the five CFIR domains: intervention characteristics, outer setting, inner setting, and characteristics of individuals to report the findings (Table 4). The first domain describes the content of the HIVST distribution models, the second and third describe the context in which the models were implemented, while the fourth describes distributor characteristics. The domains and corresponding constructs were selected deductively based on their likely influence on implementation of the distribution models. The fifth domain, process of implementation, was dropped using this criteria and because it is adequately covered in the first domain.

**Table 4:** CFIR domains

	**CFIR domain and constructs**
1	**Intervention characteristics:** features of an intervention that may influence implementation success. These include its perceived internal or external origin, evidence quality and strength, relative advantage, adaptability, trialability, complexity, design quality and presentation, and cost
2	**Outer setting:** external influences on intervention implementation. These include patient needs and resources (and barriers and facilitators to meet these needs); cosmopolitanism, or the level at which the implementing organisation is networked with other organisations; peer pressure (pressure to implement); and external policies and incentives
3	**Inner setting:** features of the implementing organisation. These include team or organisational culture, compatibility and relative priority of the intervention, structures for goal setting and feedback, leadership engagement, and the implementation climate
4	**Characteristics of individuals:** individuals' beliefs, knowledge, self-efficacy, individual stage of change, and personal attributes that may affect implementation

Sources: Nadia Safaenili et al. & Laura J Damschroder et al. [33]

## Results (lessons learned)

We first present number of HIVST kits distributed to contextualize the lessons learned. Table 5 shows kits distributed by model, and Table 6 by age group. The number of kits distributed increased over the course of the intervention period from January–December 2018 (12 months) and January -August 2019 (8 months). This increase may be attributed to people becoming aware of the availability of the kits, as well as the improvements arising from adaptations in the distribution process. Most kits were distributed through the community-led model, although coverage could be lower because the model targeted a larger population compared to the other models. Uptake of HIVST was higher among people aged 25 years and older, with very few kits distributed to adolescents aged < 19 (see Table 6).

**Table 5:** HIVST kits distributed by distribution model (from one health facility/catchment area)

Distribution model^a^	2018; Jan-Dec	2019; Jan-Aug	Total
Secondary distribution (ART)	29	139	168
Secondary distribution (ANC)	80	484	564
Community-led	1,866	3,111	4,977
Totals	1,975	3,734	5,709

^a^The workplace distribution model has not been included because distribution data was not available for some months. The community-based model using CBDAs distributed 14,625 kits in 2018 in this community

**Table 6:** Secondary distribution by age group (from one health facility/catchment area)

Age group	ART		ANC		Totals
	2018	2019	2018	2019	
Under 19 years	0 (0%)	0 (0%)	4 (5%)	42 (9%)	46 (8%)
19–24 years	0 (0%)	11 (14%)	16 (20%)	183 (38%)	210 (28%)
25 years and older	29 (100%)	128 (86%)	60 (75%)	259 (53%)	476 (64%)
Total	29 (100%)	139 (100%)	80 (100%)	484 (100%)	732 (100)

We have used the first CFIR domain, intervention characteristics, to describe characteristics of the four HIVST distribution models in the methods section. We use the remaining three domains (outer setting, inner setting, and characteristics of individuals) to report our results. Combined, the four CFIR domains explore and describe the contextual factors which influenced implementation of the models. Outer setting factors comprised compensation and incentives, stock outs and responsible organisational policy and practices. Inner setting factors included location and spread of distribution points, workload (incentives and rewards), locally available resources (communication and space), while trust in distributors and distributor typology were important individual characteristics. We illustrate flexibility and responsiveness to adapt by describing changes that were made during implementation.

### Outer setting factors

#### Compensation and incentives

Some distributors wanted to be compensated for loss of time when drawn away from their personal activities including businesses. One distributor observed that only money would truly motivate distributors. The secondary and community-led models offered material incentives, and the latter also gave out monetary incentives, while none were given out in the workplace model. The community-led and workplace models experienced high distributor attrition early on, contributing to uneven geographical distribution of *tuntemba* in the community. Community-led distributors who remained complained not only about inadequate incentives but that they were left out-of-pocket by their participation. Consequently, the transport refund, and the minimum number of kits to collect per request were increased, resulting in a reduction of trips distributors made to the health facility. However, distributors still complained about ‘broken promises’ regarding increases in monetary incentives and late payments. New distributors said they had not been given t-shirts and bags, items they noted would be useful in promoting HIVST. Incentives did not seem to matter much in the secondary distribution models since distributors were already volunteers at the health facility benefitting from other programmes. The workplace model was the most demanding for distributors and few employees were willing to become workplace champions because they would not be compensated.

#### Stock outs

Interruptions to the supply of HIVST kits were experienced until the third and seven months following distribution for the community-led and secondary distribution models, respectively. For the latter, stockouts were mainly due to poor record keeping at the facility and failure to request kits on time. On a few occasions, HIVST kits were available at the health facility but could not be found because of the disorder in the storeroom and inadequate communication between departments:

“We have had cases were test kits are there, but they call to report that they have run out due to lack of communication within departments. Sometimes you find that the box with test kits is pushed in a corner then another person comes and puts another on top and around it. By the time test kits are needed, the box can’t be seen”, (IDI, secondary distribution, implementing partner)

Stock management improved when the reporting and ordering function was reorganised and transferred from the ANC (volunteers) to the pharmacy department.

Stock outs in the community-led model occurred because distributors were only allotted 25 HIVST kits per month – an amount that did not meet the high demand. Consequently, distributors made frequent orders, putting pressure on, and slowing down the supply chain system. The quota was increased to 50 kits and eventually, the community-based distributors were allowed to order kits anytime if they accounted for the last allocation. This mitigated against stock outs in the community-led and secondary distribution models. HIVST distribution in phase one of the workplace model was done by SFH and on demand, usually consisting of a one-day activity with no stock outs.

#### Responsive organisational policy and practices

The presence or absence of HIV workplace practices and policy impacted implementation of HIVST as did related factors, such as support for workers, promotion of workers’ rights and stigma mitigation. Health activities facilitated by the workplace policy included testing for HIV, dispensing ART at the company clinics, counselling, condom distribution, and health talks for employees. Few organisations offered all the services. Some organised health talks or fairs and offered HIV testing alongside screening for hypertension, diabetes, and prostate cancer awareness. Others had health insurance, while others had online or face-to-face support and counselling services. However, some workers were skeptical of the services’ capacity to ensure total confidentiality as a stigma mitigation measure.

Organisations with a functioning and well disseminated HIV workplace/wellness policy were more likely to accept HIVST and facilitate rapid integration. HIVST distribution started late in the mine because the HIV workplace policy was practically non-existent. All permissions were supposed to be obtained from the chief executive, which delayed implementation. In contrast, the manufacturing company had a functioning HIV workplace policy that workers were aware of. HIVST distribution was easily incorporated into ongoing activities. Similarly, all the phase one organisations that invited SFH to distribute HIVST kits had HIV policies and wellness activities with designated staff, spaces for HIV testing, as well as staff support structures. Most provided other HIV services such condom distribution and information giving.

### Inner setting factors

#### Location and ‘spread’ of distribution points

HIVST clients described a well-located distribution point as being easily accessible with adequate space. The community-led model was seen as bringing services closer to the people and conveniently located: *“Where we community workers live, where people collect, it’s not as far as them coming to the clinic to queue up. So, it’s time saving”,* (FGD, community-led, distributors). In the workplace, some distribution points were located far from busy departments. Observer notes revealed that the production department of the manufacturing company was located far from the closest distribution point and yet it was the largest department; the distribution point in the mine was 1.5 km from the nearest department and 5km from the biggest department. Workers felt discouraged walking long distances, and those who could not leave their workstations unattended had few opportunities to test. “*Some workers have complained that this place is too far; I also struggle to go to the other side because of distance, imagine someone just coming here for a test kit”*, (IDI, workplace, distributors). Peer educators, in the manufacturing company, took test kits to co-workers to help workplace champions reach as many workers as possible.

#### Workload and rewards

Distribution of HIVST was an additional task in all the four distribution models. Except for the ANC clinic, distributors for the other models felt outnumbered to meet demand. The t*untemba* owners were the sole distributors in the community and were occasionally overwhelmed when many clients came at once. HIVST kit distribution at the ART clinic was the most affected because there were several programmes by different organisations requiring distributors to multi-task. The nurse helped with kit distribution when distributors could not cope with the number of clients. Distributors said additional expectations, such as participating in supply chain and writing reports, overwhelmed them, and negatively impacted their work. For instance, workplace champions said they worked over lunchtime to compensate for lost time, consequently affecting their productivity, and without corresponding incentives or rewards. Consequently, one champion in the mine dropped out. *Tuntemba* owners reported feeling compelled to choose between serving customers and HIVST clients, often switching between the two. Some reported losing HIVST clients because they could not wait to be served: *“Sometimes the shop is very busy; it’s very difficult to sell and distribute test kits simultaneously. In such cases, I need someone to help me sell while I attend to the people asking for kits”,* (IDI, community-led, distributor). The choice was made harder when clients came back for more information after self-testing or sought assistance to be linked to services at the health facility.

#### Communication and space (locally available resources)

Distribution of HIVST was more rapid, cheaper, and better sustained when it leveraged existing communication forums and available space. All four models used existing formal communication forums to promote HIVST to a lesser or greater extent. Routine health talks were used in the secondary distribution models. The HCC members and *tuntemba* owners used community meetings and other forums they attended by virtue of being influential members of the community. Occupational health and safety meetings, emails, staff memos, notice boards, wellness programmes, and gatherings in the cafeteria and pubs were used in the workplace distribution model.

Space for storage of the kits was key. Kits were kept in health facility storerooms, while in the community-led and workplace models they were stored in any available rooms. Space was limited in all models except for the ANC clinic and could hardly be expanded but some distributors showed creativity by rearranging contents of available space/rooms. Use of the pharmacy for distribution of kits to ART clients not only sorted out the space challenge but also improved quality of distribution: *“[the] best place to conduct distribution is the pharmacy just like the way medication is given and staff explain how it should be taken”* (IDI, secondary distribution, user).

#### Engaged leadership

Active involvement of management at the health facilities and workplaces improved communication, practices and support related to HIVST distribution. In the secondary and community-led models, health facility management rationalised supervision of distributors and revised roles and responsibilities between distributors and HCWs intended to reduce pressure on distributors. In the workplace model, involvement of human resources and cooperate affairs departments created trust in the employees. One employee, who collected an HIVST kit, said he was motivated to do so because he was confident his employer and co-workers would not pressure him to reveal his HIV test results as this was against workplace policy: *“What I know is that you don’t have to tell your employer your HIV status. I also know that involuntary disclosure of someone else’s status is punishable by instant dismissal in this organisation. I have not seen or heard about anybody who disclosed another person’s status because people understand the implication of doing so”*, (IDI, workplace, user).

### Characteristics of individuals

#### Trust in distributors and distributor typology

The use of trusted distributors facilitated acceptance of HIVST in all four models. Trust was central to the characteristics of a ‘good’ distributor, particularly with the community-led model because of the mistrust people can have about new things, especially if championed by outsiders. Distributors leveraged existing relations and trust: *“We noticed other people going there and decided to try, and then we discovered that it (HIVST) was good”,* (FGD, community-led, users). Participants said they preferred trained, competent distributors who had ‘good standing in the community’ and that it was easy to approach such distributors for kits and go back to seek assistance to linkage to preventive and confirmatory testing services following an HIVST. Some male distributors said they had escorted fellow men to the health facilities but were unsure if they all did their confirmatory tests and started treatment. However, one man confirmed starting treatment: *“I tested positive, but I denied my results, so I came here….. to confirm the results. I was diagnosed positive, so I started treatment. The first time, I used the oral quick then tested using blood the second and third times”,* (IDI, secondary distribution, user). Most participants said they did not mind the sex and age of the distributor as long the distributor was able to communicate with clients and give relevant information. On their part, distributors said they volunteered out of altruism, even though most wanted to be compensated for lost time. An SFH staff observed how being a distributor in the community-led model enhanced one’s self-esteem and image in the community: “*They are in a sense ‘small doctors’, especially in rural areas…”,* (IDI, community-led, implementing partner).

## Discussion

The importance of understanding context has been emphasised as key to developing interventions suitable to different settings [35, 36]. We used the CFIR to identify contextual factors influencing successful implementation of four HIVST distribution models. The community-led distribution model increased coverage better than the other models for several reasons. The *tuntembas* were owned and were easily accessible by community members, and the model was well integrated with the linked health facility and received support in terms of HIVST kits stock management. When the model faced challenges, quick adaptions such as increase in transport money for distributors, and increase in the quota of kits distributors could order at a given time normalized distribution. Our findings suggest four overarching features that are key to the successful interplay of the contextual factors in implementing HIVST distribution models: 1) integration; 2) collaboration and support; 3) ownership; and 4) accessibility and sustainability.

HIVST distribution was often added to existing portfolios of work, and some distributors sometimes felt they were being pulled in many directions. Community-led distributors, for instance, were sometimes forced to choose between promoting their business or HIVST. More careful integration of HIVST distribution into existing work schedules and logistics, and existing health programmes could have improved HIVST distribution and made HIVST kits consistently available for users. Our findings show integration was essential to all models. The WHO and International Labour Organisation recommend a smooth integration of HIVST distribution into existing HIV/ AIDS health programmes, workplace wellness, occupational health and safety initiatives [37] to improve acceptance and overall implementation climate to bring implementation of HIVST to scale [38]. Similar to findings on workplace HIV testing in general, HIVST is likely to be more acceptable when other health services such as body mass index, blood glucose, blood pressure health advice are included, normalizing HIV testing in the workplace [39, 40].

High level support, networking between organisations and trusting relationships were important to the successful distribution of HIVST in all the models. The involvement of MOH and NAC quickened the implementation of the HIVST programme. Equally, buy-in from management proved the turning point for acceptance of HIVST in the private sector organisations. Management was more likely to avail organisation communication structures and space for use in the distribution of HIVST kits if they fully embraced the programme. This ensured the successful utilization of existing structures and guidelines and policies. Consequently, communication and coordination between the different players improved thereby ensuring a steady supply of HIVST kits. The start of distribution in the secondary distribution and community-led models was plagued with communication challenges because of lack of clear reporting guidelines. In the workplace model, organisations with HIV services and wellness activities were either more likely to invite or to accept implementers’ requests to distribute HIVST kits in their workplace. Although not many organisations in the private sector have HIV/AIDS policies, policy translation is nonetheless very high [41] and HIVST programmes should leverage this to actively engage relevant stakeholders to increase uptake of HIVST.

Involvement of community gatekeepers such as the HCC and others catalyzed implementation of the community-led and to some extent the secondary distribution models, providing some level of community ownership of the models even though attrition challenges affected the initial momentum. Our findings endorse findings elsewhere that show engagement of key individuals and organisations involved in intervention delivery and oversight is critical [42, 43] for buy-in, maintenance of good communication and building trusting relations. Similar distribution models such as the peer-led model have emphasised the role of trust and relationships. For instance, community members find it easier to accept HIVST kits from someone they live with, who is regularly available and is approachable [44]. Similar to other studies, our findings show that communities can design and take ownership of the community-led HIVST distribution model [45].

Our findings confirm existing literature that individual distributors must be influential [46] and trustworthy, [47] and that distribution points must be strategically located [48] to increase accessibility of HIVST. These attributes, we believe, would be important in determining how distribution models are accessed, scaled up and sustained in different settings. In addition, sustainability would require strong leadership and support, availability of financial and human resources and provision of incentives to distributors. The community-led and workplace models particularly required strong leadership to initiate and build implementation momentum. Both financial and human resources are key for the sustainability of the models, and have been found to increase volunteer motivation [45, 49]. The WHO and ILO observe that adequate human and financial resources are required to successfully implement the workplace distribution model [37]. This observation is true of other distribution models [50]. However, our study has shown that community-led and secondary distribution models can benefit from a careful integration into the health facility HIV testing and outreach activities. A symbiotic relationship between the two distribution models would leave the community-led model less exposed to external risks, and the decisions of donor funding and increase the model’s chances of sustainability. Provision of incentives and compensation for loss of time should be considered for all distribution models, particularly for the community-led and secondary distribution models.

### Limitations

Evaluation of the secondary distribution, community-led and the first phase of the workplace distribution models started when HIVST distribution had already commenced for these models. The evaluation period of the second phase of the workplace distribution model was very short and was done when implementation of the model was in nascent stage. While this was a missed opportunity to learn about how the models were iterated throughout implementation, findings of this study are derived from real world experience and are likely to be useful for others implementing HIVST distribution at scale. Additional quantitative data, including demographic details of participants, would have provided a broader understanding of how context and/or demographic factors affected implementation and distribution.

## Conclusion

HIVST is an effective intervention, but success varies based on contextual factors and quality implementation which include having enough, motivated, and trained distributors who know their roles, availability of HIVST kits when needed, there being enough distribution points to increase access to HIVST kits and improve distribution, and an enabling environment through existence and application of relevant policies and practices that support HIVST distribution. Use of the CFIR helped to highlight how these factors mediated each other and influenced implementation and iteration of the models. Changes or adaptions to implementation such as use of existing communication channels to promote and distribute HIVST kits proved critical.

## Data Availability

Th dataset generated and / or analyzed during the current study are not publicly available due to confidentiality concerns but are available from the corresponding author upon reasonable request and with permission from the STAR consortium.

## Abbreviations

HIVST: HIV-self-testing
HIV: Human Immunodeficiency Virus
ANC: Antenatal Care
ART: Antiretroviral Therapy
WHO: World Health Organisation
STAR: Self-Testing Africa
SSA: Sub Saharan Africa
MOH: Ministry of Health
SFH: Society for Family Health
NAC: National AIDS Council
ILO: International Labour Organisation
HCC: Health Centre Committee
IDI: In-Depth Interview
FGD: Focus Group Discussion
CFIR: Consolidated Framework for Implementation Research

## Footnotes

1 A Qualitative Research Network is a collection of researchers from multiple-country studies and contexts, contributing to a wider research agenda.
